# Sexual Violence Among Higher Education Students in the United Kingdom: Results from the Oxford Understanding Relationships, Sex, Power, Abuse and Consent Experiences Study

**DOI:** 10.1177/08862605231212167

**Published:** 2023-11-20

**Authors:** Bridget Steele, Michelle Degli Esposti, Pete Mandeville, David K. Humphreys

**Affiliations:** 1 University of Oxford, UK

**Keywords:** community violence, dating violence, domestic violence, sexual assault

## Abstract

Sexual violence (SV) experienced by higher education students is a prevalent public health problem. Collecting data on SV through self-report surveys in higher education institutions (HEIs) is essential for estimating the scope of the problem, the first step to adequately resourcing and implementing prevention and response programming and policies. However, in the United Kingdom, data is limited. We used data from the cross-sectional Oxford Understanding Relationships, Sex, Power, Abuse and Consent Experiences survey, administered to all students at a university in the United Kingdom in May 2021 (*n* = 25,820), to estimate the past year prevalence of SV. We analyzed data from respondents who answered at least one question on SV (*n* = 1,318) and found that 20.5% of respondents experienced at least one act of attempted or forced sexual touching or rape, and 52.7% of respondents experienced at least one act of sexual harassment (SH). We found that women experienced the highest rates of SV. Attempted forced sexual touching was far more common than forced sexual touching, or rape. Sexist remarks or jokes were the most common act of SH. Most acts of SV took place at the university. These findings reveal that the prevalence of SV in HEIs in the United Kingdom could be far higher than what is experienced in the general population. While this study reflects the context in only one institution, it underlines the need for continued monitoring to develop rigorous, evidence-based, and targeted prevention and response strategies.

## Introduction

Sexual violence (SV) is any attempted or completed non-consensual sexual experience, including rape, sexual assault, sexual coercion, sexual aggression, and sexual harassment (SH) (Garcia-Moreno et al., 2012). SV experienced by higher education students is a pressing public health problem and can have long-lasting, detrimental consequences on survivors’ physical health and mental well-being (Abrahams et al., 2014; Black et al., 2016; Dilip & Bates, 2021; Krebs et al., 2007; McMahon et al., 2019), as well as on their educational outcomes (Baker et al., 2016; Molstad et al., 2021). Collecting SV data through self-report surveys in higher education institutions (HEIs) is essential for estimating the scope of the problem—the first step to adequately resourcing and implementing prevention and response programming and policies. Chronic underreporting of SV to university administration or law enforcement means that these institutions are limited in their ability to accurately estimate the scale of the issue (Sinozich & Langton, 2014). Collecting self-reported survey data on SV among higher education students offers HEIs the opportunity to ascertain more accurate prevalence rates, and is specifically important for (a) assessing who is impacted and what types of SV are most common; (b) monitoring trends in SV prevalence overtime; and (c) using student voices to inform institutional policies and procedures (Krause et al., 2018).

Surveys on SV among higher education students, often called “campus climate surveys,” are common in the U.S. (Fedina et al., 2018; Krause et al., 2018) as a result of Federal policies (e.g., Title IX) compelling universities to public report prevalence statistics to prospective students. But such surveys are less common globally as was found in a recent systematic review and meta-analysis on the prevalence of sexual assault (SA) (including forced sexual touching, coercive sex, attempted rape, and rape) in higher education (Steele et al., 2023). The results estimated that 18% of women, 8% of men, and 18% of transgender or gender-diverse students have experienced SA in higher education (Steele et al., 2023). This review, consistent with other reviews on campus climate surveys in the United States (Fedina et al., 2018; Krause et al., 2018), found that forced-sexual touching was the most common type of SA experienced, followed by coercive sex, attempted rape, and rape. In a review specifically on SH prevalence experienced by HEI students, Bondestam and Lundqvist (2020) found that on average one in four female students experienced unwanted conduct of a sexual nature. In addition to finding a dearth of evidence outside of the U.S. context, these reviews have reported methodological flaws in the evidence base, with many studies using unvalidated SV measures and/or having poor response rates, inadequate sampling strategies, and small sample sizes, which could bias estimates (Bondestam & Lundqvist, 2020; Steele et al., 2023).

With increasing demand for rigorous empirical research on SV at HEIs outside of the U.S., there is potential for HEIs, globally, to build on and adapt existing tools developed for U.S. campus climate surveys for measuring and responding to institutional SV (Bull et al., 2022; Humphreys & Towl, 2022; Steele et al., 2020). This is particularly relevant in the UK context (Bull et al., 2022; Phipps & Smith, 2012). Data from the UK Office for National Statistics (2021) has found that full-time students over the age of 16 experienced the highest rates of past-year SA between 2018 and 2020, when compared to people in any other occupation; across all occupations, 2.9% of women and 0.7% of men experienced SA but 11.6% of full-time female students and 4.3% of full-time male students experienced SA. Given the heightened risk students in the UK seem to face for experiencing SV and the presence of “lad culture” in UK HEIs, which normalizes sexism, SH, and violence means (Jackson & Sundaram, 2020), UK HEIs are increasingly being called upon to investigate the prevalence, nature, and culture of SV among their students (Bull et al., 2022).

In 2016, at the request of the Universities Minister, Universities UK (UUK) published their *Changing the Culture* report and found no comprehensive data on SV at universities in the UK (2016). Since then, UUK has encouraged institutions to actively draw on academic expertise and related resources to collect reliable and transparent data on SV (Bull et al., 2022). However, to date, very limited data collection has occurred and only two peer-reviewed articles have been published (Bull et al., 2022). First, Roberts et al. (2022) conducted a study among 1,034 students at an HEI in the North of England and reported on prevalence rates of unwanted sexual contact (15.9% for women and 5.4% for men) and sexual comments (32.6% for women and 9.2% for men). These rates are comparable with estimates from the *Hidden Marks* survey run by the National Union of Students in 2010, which reported that 14% of the 2,000 women respondents experienced SA or physical violence while attending an HEI (Smith, 2010). A limitation of these surveys is that they did not include a validated tool for measuring SV, instead the authors created their own questions. Further, Roberts et al. (2022) did not report the recall or time period to which the questions on SV were referring (e.g., “past year” or “as a student”).

Second Anyadike-Danes et al. (2022), conducted a study with 1,033 Northern Ireland HEI students. Anyadike-Danes et al. (2022) used the Sexual Experiences Survey (Koss & Gidycz, 1985), a valid tool for measuring SV in the higher education context, and found considerably higher rates of SV (67% of women and 44% of men respondents had an unwanted sexual experience since attending university) than Roberts et al., (2022). The estimates from Anyadike-Danes et al. (2022) were also higher than estimates from a student government-run survey, also conducted in Northern Ireland (Haughey et al., 2016). Haughey et al. (2016) found that among 3,097 respondents, 33.5% of experienced unwanted touching, and 33.2% experienced unwanted verbal SH.

The heterogeneity in estimates presented in existing studies is likely due to inconsistencies in how SV was defined, measured, and reported on in these studies and the use of different sampling strategies across the studies, which have implications for the validity and reliability of the findings. None of these studies have accounted for, or explored, the implications of potential of nonresponse bias related to experiencing or not experiencing SV. Additionally, these studies did not report on transgender or gender-diverse samples, or statistically test for differences in experiences of SV across respondent characteristics. As a result, the prevalence of SV and which students are most at risk in HEI settings in the UK is still poorly understood.

In this study, we advance the evidence on SV at UK HEIs by using the Oxford Understanding Relationships, Sex, Power, Abuse and Consent Experiences (OUR SPACE) survey data—a rigorous cross-sectional study of all undergraduate and graduate students over the age of 18 at a large UK university in the South West of England—to provide an estimate of the past year prevalence of SV experienced by HEI students at a UK HEI disaggregated by gender identity, sexual orientation, ethnicity, student status, and location of the incident.

## Methods

### Procedure

We used data from the cross-sectional OUR SPACE survey (for study protocol see: Steele et al., 2021) administered at a university in the UK in 2021. The OUR SPACE survey was administered to all students at the university (*n* = 25,820), via email, in May 2021. The link to complete the survey was open for 2 months after the launch and students received three reminder emails. Direct emails to students with the survey link were supplemented by additional online promotional campaigns run through the student government, colleges, and university platforms, to raise awareness of the survey and to encourage participation in the study.

The electronic survey was implemented using Qualtrics™ survey software. Respondents opted in via informed consent. All responses were anonymous and confidential and the Qualtrics survey platform ensured that responses were encrypted. The survey took on average 15 min to complete and respondents were able to skip any question at any point during the survey to help prevent re-traumatization. Respondents were also able to terminate the survey at any time. Students who participated were provided with a list of supports and resources before, during, and after completing the survey. Those who completed the survey indicated if they wanted to be entered into a lottery to receive a prize (iPads [3] and £50 Amazon vouchers [10]). Ethical approval was obtained from The University of Oxford (Ref No: R73805/RE001).

### Sample

**Figure 1.**
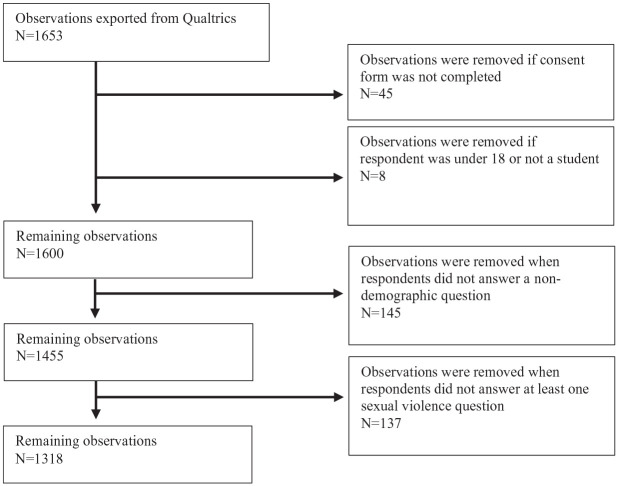
Our space data flow chart.

There were 1,600 students who responded to the survey (approximately 6% of the target population) and 1,455 students who answered at least one non-demographic question. Respondents were able to skip any question on the survey, aligning with best practice research ethics for online surveys (Buchanan & Hvizdak, 2009). Consistent with guidance from previous validation studies (Cantor et al., 2015; Krebs et al., 2016), we analyzed data from respondents who answered at least one question on SV (*n* = 1,318), which comprised approximately 5% of the student population (see Figure 1).

### Measures

Survey measures were adapted from previously conducted campus climate surveys, specifically the Johns Hopkins University *Health and Safety Study* from 2017, and psychometric questions from the Sexual Experiences Survey (SES) (Koss et al., 2007). See Table 1 for a description of the survey items used in the analyses. We collected data on a range of SV acts, including SH. SH is a subset of SV that includes unwanted comments, advancements, or propositions that are implicitly or explicitly sexual in nature but do not involve attempted or completed physical touch (Fitzgerald, 1990). While SH sits within SV, it is at times important to distinguish between SH and other forms of SV such as SA, including attempted and completed sexual touching and rape to better understand the experiences of survivors. Therefore, for this study, SH is measured distinctly from other forms of SV.

**Table 1.** Description of Measures Used in the Oxford Understanding Relationships, Sex, Power, Abuse and Consent Experiences Survey.

Topic	Question and Response Options	Tool Used
Gender identity	Do you identify as: • A cisgender man • A transgender man • A cisgender woman • A transgender woman • Gender non-conforming, nonbinary, or queer • None of the above	UK Office for National Statistics (2021)
Student status	Are you registered as a: • Full-time undergraduate student • Full-time postgraduate student • Visiting student • Continuing education student • Part-time undergraduate student • Part-time postgraduate student	University of Oxford Student Data Management and Analysis (2021)
Ethnicity	Do you identify as: • Arab • Asian/Asian British • Black/African/Caribbean/Black British • Mixed/multiple ethnic groups • White • Any other ethnic group	UK Government (2021)
Sexual orientation	What is your sexual orientation? • Bisexual • Gay or lesbian • Heterosexual • None of the above	UK Office for National Statistics (2021)
Sexual harassment	Please indicate if you have experienced any of the following in the past year either in person or online (select all that apply). • Someone made a sexist remark or sexist joke about me in my presence • Someone made an inappropriate comment about my body, appearance, or attractiveness, in my presence • Someone said crude or gross sexual things to me or about me • Someone has repeatedly asked me to go on dates or spend time with them even after I said no • Someone stalked, followed, or repeatedly contacted me when I did not want them to	Johns Hopkins University *Health and Safety Study* (Campbell et al., 2017)
Sexual assault	Have you experienced any of the following in the past year? Please select the box next to acts that you have experienced in the past year. • Someone tried to fondle, kiss, or rub up against the private areas of my body or tied to remove some of my clothes even though I didn’t want that • Someone fondle, kiss, or rub up against the private areas of my body or removed some of my clothes even though I didn’t want that • Someone tried to sexually penetrate me (someone tried to put a penis or insert fingers or objects into my vagina or anus) even though I didn’t want that • Someone sexually penetrated me (someone put a penis or insert fingers or objects into my vagina or anus) even though I didn’t want that • Someone tried to perform oral sex on me or make me perform oral sex even though I didn’t want that • Someone performed oral sex on me or made me perform oral sex even though I didn’t want that	Adapted from the Sexual Experiences—Short Form Victimization (Koss et al., 2007)

### Analyses

Descriptive frequencies were calculated to estimate the proportion of the student population to have experienced any act of SV in the reporting period (past year). The past year prevalence for each of the acts of SA and SH was calculated, treating each outcome as a binary variable (Spronk et al., 2019). For total SA, those who reported not experiencing any SA act were coded as 0, while those who reported one or more experiences of SA were coded as 1. The same process was applied for calculating the total SH prevalence. Confidence intervals of prevalence estimates used the Wilson score interval, which is appropriate for small sample sizes (Brown et al., 2001). Occurrences were stratified by sociodemographic groups (ethnicity, gender identity, sexual orientation, and student status), using cross-tabulations and Chi-square statistics to compare and test for significant differences across subgroups. The location where the most recent incident of each type of SA and SH occurred was also explored using descriptive statistics.

To help mitigate the potential of nonresponse bias, the OUR SPACE survey data was weighted using available information on the entire student population. Weighting is a statistical technique implemented after survey data have been collected to improve the accuracy of estimates from survey samples that are not representative of the total population (Lavallée & Beaumont, 2015). Weights for the OUR SPACE data were derived from publicly accessible yearly student statistics data published by the institution. An iterative proportional fitting method (often referred to as raking) was used to apply the weights (Kalton & Flores-Cervantes, 2003). To weigh the data, we chose a set of variables where the marginal population proportions were known. The December 2020 demographic data was used as it best estimated the total population of the university at the time the OUR SPACE survey was disseminated. The following variables were selected: student status (undergraduate student, postgraduate student, visiting student, or continuing education student); ethnicity; fee status (domestic or UK student, EU student, or international non-EU student), and gender/sex. Weights were then iteratively adjusted for each case until the sample distribution aligned with the population for the chosen variables. Finally, the analyses used on the unweighted data were repeated with the weighted data.

The weighted data helped to understand how the prevalence estimates might look if the sample from the OUR SPACE data was more representative of the total university population. However, there was still the potential for response bias related to experiencing or not experiencing SA or SH. Students may have had a difference in propensity to respond to the survey if they had been exposed to SA or SH. It was not possible to know what the prevalence rate was among non-respondents, and given our sample size we deemed it important to consider the implications of the prevalence rates we report. Estimates were calculated for different scenarios of potential prevalence among the non-respondents to show the possible ways in which non-response bias could change the prevalence under different assumptions. In doing so we followed best practice guidance on being explicit about the assumptions made in our analyses and guidance from Rosenberg et al., 2019 who conducted a similar analysis. Rosenberg et al. (2019) first developed this analysis to quantify the impact of nonresponse bias in campus climate surveys. They simulated the impact different scenarios, where the prevalence of SV among nonrespondents differed from responders, would have on the estimated total population prevalence of SV. Using the analysis developed by Rosenberg et al. (2019), we aimed to estimate the uncertainty of prevalence estimates from the OUR SPACE survey. To do this, we first estimated the total population prevalence using the two most extreme scenarios of prevalence among nonrespondents (all nonrespondents did experience SA or SH and all nonrespondents did not experience SA or SH). We then estimated the total population prevalence using simulations where the prevalence of SA and SH differed between responders and nonrespondents by ±5%, ±10%, and ±15% (Rosenberg et al., 2019).

All statistical analyses were conducted in R (version 3.5.1) using the survey package (v4.0; Lumley, 2020).

## Results

### Sample Characteristics

Within the sample of 1,318 students, women were over-represented with 58.6% identifying as a woman, but only 35.7% as a man (see Table 2 for full sample characteristics). The sample also included 4.4% who identified as transgender or gender diverse. The sample was also over-represented with undergraduate students (59.3%) and under-represented with graduate students (38.6%). The vast majority of students identified as White (78.7%). The next most common ethnicity was Asian or Asian British, followed by mixed or multiple ethnic groups, which comprised of 10.8% and 6.1% of the sample, respectively. Over half of the sample identified as heterosexual (58.7%) but there was also substantial representation for those identifying as bisexual (21.8%) and as gay or lesbian (8.0%).

**Table 2.** Sample Characteristics.

Variable	Unweighted	Weighted
	*N* = 1,318	*N* = 1,318
Gender		
A man	470 (35.7%)	661 (50.2%)
A woman	773 (58.6%)	637 (48.3%)
Transgender or gender-diverse	58 (4.4%)	—
Prefer not to say	17 (1.3%)	20 (1.5%)
Ethnicity		
Asian/Asian British	142 (10.8%)	268 (20.3%)
Black/African/Caribbean/Black British	26 (2.0%)	41 (3.1%)
Mixed/Multiple ethnic groups	80 (6.1%)	82 (6.2%)
White	1,037 (78.7%)	879 (66.7%)
Any other ethnic group	30 (2.3%)	41 (3.1%)
No answer	3 (0.2%)	6 (0.5%)
Student status		
Continuing education student	18 (1.4%)	51 (3.9%)
Postgraduate student	509 (38.6%)	597 (45.3%)
Undergraduate student	781 (59.3%)	656 (49.8%)
Visiting student	9 (0.7%)	14 (1.0%)
No answer	1 (<0.1%)	1 (<0.1%)
Sexual orientation		
Bisexual	287 (21.8%)	234 (17.8%)
Gay or Lesbian	106 (8.0%)	110 (8.4%)
Heterosexual	774 (58.7%)	845 (64.1%)
None of the above	72 (5.5%)	59 (4.5%)
No answer	79 (6.0%)	70 (5.3%)

### SA Experienced in Past Year

Between May 2020 and 2021, 20.5% (95% CI [18.4%, 22.7%]) of respondents experienced at least one act of SA. The most common type of SA was attempted unwanted sexual touching, followed by unwanted sexual touching. Attempted or completed rape were the least common types of SA (Table 3). Results based on the weighted data described similar, yet slightly attenuated, prevalence rates. Overall, a marginally reduced percentage of respondents experienced SA in the weighted data, whereby 17.7% (95% CI [15.7%, 19.8%]) of respondents who experienced at least one act of SA; see Table 3.

**Table 3.** Types of Sexual Assault Experienced by Respondents.

Types of Sexual Assault	Unweighted *N* = 1,318		Weighted *N* = 1,318	
	Number of Respondents	Prevalence [95% CI]	Number of Respondents	Prevalence [95% CI]
Any type of sexual assault	270	20.5% [18.4, 22.7]	233	17.7% [15.7, 19.8]
Attempted unwanted sexual touching	205	15.6% [13.7, 17.6]	176	13.4% [11.6–15.3]
Unwanted sexual touching	150	11.4% [9.8, 13.2]	128	9.7% [8.2–11.4]
Attempted rape	76	5.8% [4.6, 7.2]	62	4.7% [3.7–6.0]
Rape	76	5.8% [4.6, 7.2]	56	4.2% [3.3–5.5]

*Note.* CI = Confidence interval.

SA was experienced differently based on participant characteristics. Women (26.9%, 95% CI [23.8%, 30.0%] and transgender and gender diverse respondents (17.2%, 95% CI [9.6%, 28.9%], experienced the highest rates of SA (Table 4). Women experienced significantly higher rates of SA than men (10.6%, 95% CI [8.2%, 13.8%]), χ^2^(1, *N* = 1,243) = 45.4, *p* < .0001. While transgender and gender-diverse respondents experienced more SA than men, the difference was not significant, χ^2^(1, *N* = 528) = 1.63, *p* = .2021. There was also no significant difference in SA found between non-White (19.1%, 95% CI [14.9%, 24.1%]) and White students (26.5%, 95% CI [23.6%, 29.6%]) χ^2^(1, *N* = 1,315) = 0.4, *p* = .5. However, participants who identified as gay, lesbian, or bisexual, experienced significantly higher rates of SA (27.3%, 95% CI [23.5%, 31.5%]) when compared to heterosexual respondents (16.5%, 95% CI [14.1%, 19.3%]), χ^2^(1, *N* = 1,239) = 20.7, *p* < .0001. Repeating these tests using the weighted data did not change the significance of the results.

**Table 4.** Sexual Assault Prevalence Disaggregated by Respondents’ Characteristics.

Variable	Unweighted	Weighted
	*N* = 270	*N* = 233
Gender		
A man	50 (18.5%)	68 (29.2%)
A woman	207 (76.7%)	161 (69.1%)
Transgender or gender-diverse	10 (3.7%)	—
Prefer not to say	3 (1.1%)	4 (1.7%)
Ethnicity		
Asian/Asian British	24 (8.9%)	39 (16.7%)
Black/African/Caribbean/Black British	4 (1.5%)	4 (1.7%)
Mixed/Multiple ethnic groups	18 (6.7%)	16 (6.9%)
White	217 (80.4%)	165 (70.8%)
Any other ethnic group	7 (2.6%)	10 (4.3%)
Student status		
Continuing education student	3 (1.1%)	9 (3.9%)
Postgraduate student	71 (26.3%)	73 (31.3%)
Undergraduate student	194 (71.9%)	149 (63.9%)
Visiting student	2 (0.7%)	2 (1.0%)
Sexual orientation		
Bisexual	92 (34.1%)	42 (18.0%)
Gay or Lesbian	25 (9.3%)	20 (8.4%)
Heterosexual	128 (47.4%)	149 (64.0%)
None of the above	11 (4.1%)	7 (3.2%)
No answer	14 (5.2%)	12 (5.3%)

Undergraduate students, who were on average younger than graduate students, in the sample experienced significantly higher rates of SA (24.8%, 95% CI [21.9%, 28.0%]) than graduate students (14.0%, 95% CI [21.9%, 28.0%]), χ^2^(1, *N* = 1,290) = 21.7, *p* < .0001, however the significance of this finding diminished when using the weighted data.

### SH Experienced in Past Tear

We found that SH was more pervasive than SA. Between May 2020 and 2021, 52.7% (95% CI [50.0%, 55.3%]) of respondents experienced at least one act of SH. The most common types of SH were sexist remarks, followed by inappropriate comments made about appearance and crude sexual comments or jokes. Persistent requests to go on dates after saying no and being stalked were the least common types of SH (Table 5). Within the weighted sample, we found slightly smaller prevalence rates, whereby 47.2% (95% CI [44.5%, 49.9%]) respondents experienced at least one act of SH; see Table 5.

**Table 5.** Types of Sexual Harassment Experienced.

Types of Sexual Harassment	Unweighted *N* = 1,318		Weighted *N* = 1,318	
	Number of Respondents	Prevalence [95% CI]	Number of Respondents	Prevalence [95% CI]
Any type of sexual harassment	694	52.7 [50.0, 55.3]	621	
Sexist remark	478	36.3% [33.7, 38.9]	392	29.7% [27.3, 32.3]
Inappropriate comments about appearance	475	36.0% [33.5, 38.7]	421	31.9% [29.5, 34.5]
Crude sexual comments or jokes	306	23.2% [21.0, 25.6]	271	20.6% [18.5, 22.8]
Persistent requests to going on dates after saying no	192	14.6% [12.8, 16.6]	163	12.4% [10.7, 14.3]
Being stalked	188	14.3% [12.5, 16.3]	159	12.1% [10.4, 13.9]

*Note.* CI = Confidence interval.

SH, like SA, was also experienced differently across different student subgroups (Table 6). Women and transgender and gender-diverse respondents experienced significantly more SH than men. The prevalence of SH for women (65.8%, 95% CI [62.4%, 69.2%]) was more than double the SH experienced by men (31.3%, 95% CI [27.3%, 35.6%]), χ^2^(1, *N* = 1,243) = 139, *p* < .0001. Transgender and gender-diverse respondents, experienced lower amounts of SH (53.5%, 95% CI [40.8%, 65.7%]) than women but significantly more SH than men, χ^2^(1, *N* = 528) = 10.4, *p* = .001. Further, almost two-thirds of non-heterosexual respondents (62.8%, 95% CI [58.3%, 67.1%]) experienced SH which was significantly more than heterosexual respondents (46.4%, 95% CI [42.9%, 49.9%], χ^2^(1, *N* = 1,301) = 30.7, *p* < .0001). However, there was no difference in SH experiences between non-White (49.6%, 95% CI [43.8.4%, 55.5%]), and White respondents (53.4%, 95% CI [50.4%, 56.4%]). Further, undergraduate students experienced similar rates of SH (54.0%, 95% CI [50.5%, 57.5%]), as graduate students (51.1%, 95% CI 4[6.8%, 55.4%]), χ^2^(1, *N* = 1,290) = 1.0, *p* = .3. The results remained the same when the tests were repeated on the weighted data.

**Table 6.** Sexual Harassment Prevalence Disaggregated by Respondent Characteristics.

Variable	Unweighted	Weighted
	*N* = 694	*N* = 621
Gender		
A man	147 (21.2%)	209 (33.7%)
A woman	509 (73.3%)	403 (64.9%)
Transgender or gender-diverse	31 (4.5%)	—
Prefer not to say	7 (1.0%)	10 (1.6%)
Ethnicity		
Asian/Asian British	62 (8.9%)	106 (17%)
Black/African/Caribbean/Black British	14 (2.0%)	21 (3.4%)
Mixed/Multiple ethnic groups	48 (6.9%)	46 (7.4%)
White	554 (79.8%)	428 (68.9%)
Any other ethnic group	15 (2.2%)	20 (7.7%)
No answer	1 (0.1%)	0 (0.0%)
Student status		
Continuing education student	5 (0.7%)	15 (2.4%)
Postgraduate student	260 (37.5%)	273 (43.9%)
Undergraduate student	422 (60.8%)	325 (52.3%)
Visiting student	7 (1.0%)	9 (1.4%)
No answer	0 (0%)	0 (0.0%)
Sexual orientation		
Bisexual	200 (28.8%)	157 (25.3%)
Gay or Lesbian	55 (7.9%)	53 (8.5%)
Heterosexual	359 (51.7%)	346 (55.7%)
None of the above	37 (5.3%)	28 (4.6%)
No answer	43 (6.2%)	37 (5.9%)

### Location of Incidents

Respondents provided the location information for a total of 553 acts of SA and 1,934 acts of SH. Almost half of the SA acts took place at the university (e.g., student accommodation, common rooms, and event spaces) and a third took place in the city where the university is located but not at the university (e.g., bars, off-campus housing, and public space). For SH, half of the acts took place at the university and almost a quarter took place in the city where the university is located but not in the university (Table 7).

**Table 7.** Location of Most Recent Act of Sexual Assault and Sexual Harassment Experienced by Respondents.

Location	Acts of Sexual Assault	Acts of Sexual Harassment
	*N* = 553	*N* = 1,934
At the university	243 (43.9%)	967 (50.0%)
In the city where the university is located	166 (30.0%)	445 (23.0%)
Outside the city where the university is located	100 (18.1%)	271 (14.0%)
Online	—	193 (10.0%)
No location reported	44 (8.0%)	39 (2.0%)

### Nonresponse Bias

Only a small portion of the total university student body responded to the OUR SPACE survey (*n* = 1,318). Most of the student population who received the survey chose not to participate (*n* = 24,502). This limited the generalisability of the OUR SPACE prevalence estimates. While weighting the data helped to mitigate the low response rate, there was still the potential that students had different propensities to respond based on their experiences of SA and SH. Therefore, we conducted a series of simulations to estimate the prevalence of SA and SH among the total population through different assumptions about the prevalence among non-respondents.

We found that the prevalence of SA and SH among responders was highly sensitive to assumptions about prevalence in nonrespondents (Table 8). Under the most extreme scenarios that no nonrespondents experienced SA or that all nonrespondents experienced SA, the prevalence among the total population was 1.0% and 95.9% respectively. Similarly for SH, the prevalence was 2.7% and 97.6%, respectively. However, these extreme scenarios are likely not realistic or plausible (Giroux et al., 2020; Jeffrey et al., 2022; Rosenberg et al., 2019). More reasonable assumptions about the prevalence of SA and SH in non-responders (Cantor et al., 2015; Giroux et al., 2020) narrowed the potential range of simulated prevalence estimates to 6.3% to 34.7% for SA and 38.5 to 66.9% for SH. For example, if nonrespondents had fewer experiences of SA and SH by 5%, 10%, or 15%, the simulated population prevalence for SA would be 15.8%, 11.0%, and 6.3%, respectively and the simulated population prevalence for SH would be 48.0%, 43.2%, and 38.5% respectively. Similarly, if the non-responders experienced higher levels of SA and SH by 5%, 10%, or 15%, the simulated population prevalence shifted to 25.2%, 30.0%, and 34.7% for SA, and 57.4%, 62.2%, 66.9% for SH.

**Table 8.** Sexual Violence Prevalence Predicted Across Nine Scenarios with Different Assumptions About Non-Respondents.

Different Assumptions Made About Prevalence in Nonrespondents^a^			Total University Student Population			
Description	Mean Prevalence (SA) (%)	Mean Prevalence (SH) (%)	Simulated Prevalence SA [95% CI]	Predicted # of Students^b^	Simulated Prevalence SH [95% CI]	Predicted # of Students^b^
No sexual violence or sexual harassment	0.0	0.0	1.0% [0.9, 1.2]	270	2.7% [2.5, 2.9]	694
−15% prevalence	5.5	37.7	6.3% [6.0, 6.6]	1,618	38.5% [37.9, 39.1]	9,931
−10% prevalence	10.5	42.7	11.0% [10.3, 11.4]	2,843	43.2% [42.6, 43.8]	11,156
−5% prevalence	15.5	47.7	15.8% [15.3, 16.2]	4,068	48.0% [47.3, 48.6]	12,381
Complete case^c^	20.5	52.7	20.5% [18.4, 22.7]	5,293	52.7% [50.0, 55.3]	13,607
+5% prevalence	25.5	57.7	25.2% [24.7, 25.8]	6,518	57.4% [56.8, 58.0]	14,831
+10% prevalence	30.5	62.7	30.0% [29.4, 30.6]	7,743	62.2% [61.6, 62.3]	16,057
+15% prevalence	35.5	67.7	34.7% [34.2, 35.3]	8,968	66.9% [66.4, 67.5]	17,282
All sexual violence and sexual harassment	100.0	100.0	95.9% [95.7, 96.2]	24,772	97.6% [97.4, 97.8]	25,196

*Note.* CI = Confidence interval; SA = sexual assault; SH = sexual harassment.

a The number of non-respondents was 24,502.

b To arrive at these numbers, the simulated prevalence proportion was applied to the total study population in the 2020 to 2021 academic year (25,820).

c Respondents and non-respondents have the same prevalence of sexual assault and sexual harassment.

## Discussion

Using the OUR SPACE data we found that one in five respondents experienced SA and almost half of respondents experienced SH in a 1-year period. Most acts of SV took place either at the university or in the city where the university was located. We found that the most common act of SA act experienced by respondents was attempted unwanted sexual touching (15.6%) and that the most common acts of SH were sexist remarks (36.3%) and inappropriate comments about someone’s appearance (36.0%). A theoretical and empirical gap exists in explaining why certain types of SV are more common, yet the implications of these findings are pertinent for prevention and response. SV encapsulates a wide range of acts with varying degrees of potential harm for survivors; however, it is often discussed as one type of experience. Knowing what types of SV are most common and where they are occurring is useful for tailoring intervention efforts.

The estimates from the OUR SPACE survey were considerably higher than the estimates from the UK Office for National Statistics (2021), but this was not surprising when we consider the emerging discussion from academic researchers working on this topic in the UK (Humphreys & Towl, 2022). Our results fell within the range of estimates found in previous campus climate surveys in the UK (Anyadike-Danes et al., 2022; Haughey et al., 2016; Roberts et al., 2022). However, it is challenging to situate the OUR SPACE prevalence estimates within existing data on SV from UK HEIs due to the lack of methodological standardization across studies. Previous studies in the UK have not consistently used the same tools to measure SV, sampling strategies, or recall period. The OUR SPACE survey employed a psychometrically validated tool and a rigorous sampling strategy, while following best practice guidance on how to administer campus climate surveys.

The pervasiveness of SV among higher education students, identified within the OUR SPACE survey, is consistent with literature showing the routine and common nature of SV in society in general (Stanko, 1990), and among HEI students specifically (Hirsch & Khan, 2020; Marshall et al., 2014; Rosenthal et al., 2016; Steele et al., 2023; Sutton et al., 2021). Our finding that SV is experienced differently across gender identities, with women experiencing the largest burden of SV, echoes existing evidence (Cusano et al., 2021; Hoxmeier, 2016; Steele et al., 2023). Seen through a critical feminist lens, SV is intrinsically linked to power dynamics based on social identities (e.g., gender identity) and systems of oppression (Wooten, 2015) (Linder, 2018) and intertwined with forms of oppression and discrimination including heteronormativity, homophobia, and racism (Harris et al., 2017). Consistent with the limited previous work we found that transgender and gender-diverse respondents also experienced high rates of attempted and completed sexual touching and rape (Cusano et al., 2021; Hoxmeier, 2016) and that non-heterosexual respondents experienced more SV than heterosexual respondents (Mennicke et al., 2021; Santelli et al., 2018).

While men experienced lower prevalence rates than women and transgender or gender-diverse people, a significant proportion of men still reported experiencing SA and SH (10.6% and 31.3% respectively). This finding contradicts traditional notions of men’s SV as “invisible and incomprehensible” (Hlavka, 2017, p. 482). Men’s experiences of SV may uniquely involve certain stigmas around shame, embarrassment, and disbelief, centered around societal constructions of masculinity, dominance, and power (Hlavka, 2017). However, like women and transgender and gender-diverse people, men’s experiences of SV cannot be understood uniformly. Men who identify as gay and bisexual have been found to experience SV at higher rates than heterosexual men (Bullock & Beckson, 2011; Donne et al., 2018). Future studies on SV in the UK HEI context could be conducted to understand men’s SV in a more nuanced way and to further contribute to the limited existing evidence base on men’s SV in HEIs.

Most research on SV in HEIs has focused on the undergraduate student population. There is limited evidence of understanding how graduate students experience SV (McMahon et al., 2021; Rosenthal et al., 2016). We found that undergraduate students experienced significantly higher rates of SA than graduate students but that the two groups experienced similar rates of SH. Established risk factors for SV in higher education such as being in first or second year (Cranney, 2015; Kimble et al., 2008), lack of experience with alcohol (Gross et al., 2006), and vulnerability as being a new student (Armstrong et al., 2006) might help explain why undergraduate students appear to face more SA. More research is needed to understand why undergraduate students in the UK context might experience more SV than graduate students. The scant existing evidence on SV among graduate studies has showed that the unique relationships graduate students form with faculty and staff at universities, when compared to undergraduate students, places them at an increased risk of experiencing SV from people in positions of power and authority (Bull & Page, 2021; McMahon et al., 2021).

A key limitation to the generalizability of the prevalence estimates is the likely presence of response bias—the potential for certain groups of people to be more likely to respond to the survey compared to others, due to the low response rate. It is likely not the case that a random selection of the student population will have responded. For example, participants may have been more likely to respond if they had experienced SV. Alternatively, participants who have experienced SV may have been deterred from responding to prevent re-traumatization. To help mitigate the issue of response bias, we weighted certain survey variables based on known total population characteristics and underwent a complete case analysis to deal with missing responses (Buskirk & Kolenikov, 2015). In addition, we conducted a series of simulations to understand the range of potential prevalence estimates under scenarios where non-respondents had higher or lower rates of SV when compared to respondents. Under realistic simulation assumptions, we still identified high prevalence rates for SA and SH of 15.8% to 25.2% and 48.0% to 57.4% respectively. Nevertheless, our weighted and simulation analyses may not fully counteract issues relating to response and non-response bias, and future campus climate surveys should aim to obtain high response rates for the entire target population.

Another limitation, to the OUR SPACE survey is that more psychometric testing is needed to understand the reliability and validity of the measure. The attempted and completed sexual touching and rape measure relies on the previously validated SES (Koss & Gidycz, 1985), but this measure does not include acts of sexual strangulation as a form of SV. Emerging evidence is showing high rates of strangulation during sexual activity among young people and subsequent iterations of the OUR SPACE survey should incorporate questions asking about this act (Edwards & Douglas, 2021; Herbenick et al., 2021). The SH measure was adapted from existing US campus climate surveys (Campbell et al., 2017) but has not been validated in the UK context. Research on SV globally tends to rely on US measures and definitions when conducting research. In subsequent versions of this survey, a revised scale of SH questions could be added and validated. This scale could include explicit wording that aligns better with the UK legal definition of SH including, incorporating questions on sexualized images, and eliminating the questions related to sex-based discrimination, which do not fall under UK legal definitions of SH. While it is important that legal definitions are considered in creating measurements of SV, it might also be important to consider a wider range of actions that fully capture the experiences of survivors of SV. For example, Bull and Page (2021) refer “boundary blurring” behaviors, often used to describe behaviors of staff toward students that students are unclear how to interpret but ultimately have harmful impacts on students.

A third limitation of the OUR SPACE survey is that measures exclusively focused on SV and there were no questions on other forms of domestic and intimate partner violence such as physical abuse, economic or financial abuse, or coercive control. In the UK, scholars and policy makers are increasingly encouraging researchers to include these types of violence in questionnaires (Bull et al., 2022; Khan, 2021; UniversitiesUK, 2020). While measuring this type of violence is important, we intentionally only focused on SV for practical and conceptual reasons. We needed to keep the survey short to increase participation rates and not place an additional burden on students. Reducing the detailed questions we had on SV to include room for questions on SV would have sacrificed the quality and rigor of our SV measurement. We also know that SV often occurs outside of traditional dating or intimate partner relationships, and it is important to account for the ‘hook up’ culture that exists among HEI students and look at violence that occurs among strangers, peers, and within casual sexual encounters. Most young adults at HEIs have engaged in sexual acts with people they are not in a committed relationship with (Duval et al., 2018; Kuperberg & Padgett, 2016; Monto & Carey, 2014). Further, evidence suggests that these casual sexual encounters, or “hookups,” are increasing at HEIs and that most committed romantic relationships at HEIs begin with a casual hookup (England et al., 2008; Garcia et al., 2012; Lambert et al., 2003). Research that exclusively focuses on SV within dating or intimate partner relationships is limited as it fails to consider sexual encounters between strangers, acquaintances, friends, and previous romantic partners where SV can and does occur (Duval et al., 2018).

Finally, the OUR SPACE survey was administered during the COVID-19 pandemic and therefore the interpretation of prevalence rates must consider that the living arrangements and social activities of respondents during the reporting period would have been impacted by government and university safety regulations.

Results from the OUR SPACE survey highlight the pervasiveness of SV among HEI students at a university in the UK, and identified certain sub-populations who are at elevated risk of victimization (e.g., women and non-heterosexual). While these findings provide critical evidence for advancing understanding of SV in HEI settings and may help to tailor prevention and response programming, continued research is needed to better understand this public health issue, including research at other HEIs with different demographic characteristics among their student body. OUR SPACE survey has the potential to become a repeated cross-sectional survey given the transparent and reproducible research practices used and the existing partnerships and interest from university administration and student groups. However, efforts need to be made to increase response rates and student engagement. Accurate prevalence estimates can play an important first step in establishing a larger research agenda across the sector and the repeat administration and monitoring will help to strengthen the OUR SPACE survey, in turn providing critical insights for tackling the evolving problem of SV at HEIs.
